# Exogenous application of ALA enhanced sugar, acid and aroma qualities in tomato fruit

**DOI:** 10.3389/fpls.2023.1323048

**Published:** 2023-12-21

**Authors:** Ruirui Li, Junwen Wang, Hong Yuan, Yu Niu, Jianhong Sun, Qiang Tian, Yue Wu, Jihua Yu, Zhongqi Tang, Xuemei Xiao, Jianming Xie, Linli Hu, Zeci Liu, Weibiao Liao

**Affiliations:** ^1^ College of Horticulture, Gansu Agricultural University, Lanzhou, China; ^2^ State Key Laboratory of Aridland Crop Science, Gansu Agricultural University, Lanzhou, China

**Keywords:** tomato, ALA, sugar-metabolism, acid-metabolism, volatile components

## Abstract

The content and proportion of sugars and acids in tomato fruit directly affect its flavor quality. Previous studies have shown that 5-aminolevulinic acid (ALA) could promote fruit ripening and improve its aroma quality. In order to explore the effect of ALA on sugar and acid quality during tomato fruit development, 0, 100, and 200 mg L^−1^ ALA solutions were sprayed on the fruit surface 10 days after pollination of the fourth inflorescence, and the regulation of ALA on sugar, acid metabolism and flavor quality of tomato fruit was analyzed. The results showed that ALA treatment could enhance the activities of acid invertase (AI), neutral invertase (NI), and sucrose synthase (SS), reduce the activity of sucrose phosphate synthase (SPS), up-regulate the expression of *SlAI*, *SlNI* and *SlSS*, change the composition and content of sugar in tomato fruit at three stages, significantly increase the content of sugars in fruit, and promote the accumulation of sugars into flesh. Secondly, ALA treatments increased the activities of phosphoenolpyruvate carboxykinase (PEPC), malic enzyme (ME), and citrate synthase (CS), up-regulated the expression of *SlPPC2*, *SlME1*, and *SlCS*, and reduced the citric acid content at maturity stage, thereby reducing the total organic acid content. In addition, ALA could also increase the number and mass fraction of volatile components in mature tomato fruits. These results indicated that exogenous application of ALA during tomato fruit development could promote the formation of fruit aroma quality and were also conducive to the formation of fruit sugar and acid quality.

## 1 Introduction

As one of the most popular vegetables, tomato (*Solanum lycopersicum*) stands out for its abundant nutritional content and distinctive flavor. At present, consumers are keen on tomatoes with the perceived “perfect taste”, making taste preference a significant factor in tomato selection and consumption (Li et al., 2018). Tomato fruit quality mainly consists of appearance quality, nutritional quality, flavor quality, processing, and storage quality (Lipan et al., 2021; Li et al., 2023). Among them, flavor quality primarily comprises sweetness (which is related to sugars in fruits), sourness (which is related to acids in fruits), and aromatic smell (which is related to volatile aromatic substances in fruits). The loss of flavor in tomato fruits can significantly impact their sensory quality, thus affecting their economic value (Maul et al., 2000).

The soluble sugar in tomato fruit primarily comes from photosynthesis and sucrose metabolism (Powell et al., 2012). Moreover, notable variations in sugar accumulation and composition exist among diverse tomato varieties and fruit samples (Lombardo et al., 2011). Sucrose synthesis and decomposition in fruit were affected by enzymatic activities, including sucrose phosphate synthase (SPS), sucrose synthase (SS), and invertase (Ivr). Sucrose synthase (SS) is classified into sucrose synthase-synthesis (SS-S) enzyme and sucrose synthase-decomposition (SS-C) enzyme due to its dual functionality in both sucrose synthesis and sucrose decomposition during different stages of plant development. Invertase (Ivr) is categorized into acid invertase (AI) and neutral invertase (NI) based on the distinct pH conditions in the fruit under which they operate (Klann et al., 1993; Yamaki, 2010). Among these enzymes, sucrose phosphate synthase (SPS) and sucrose synthase-synthesis direction (SS-S) play a role in synthesizing sucrose, thereby promoting an increase in sucrose content. On the other hand, invertase (Ivr) and sucrose synthase-decomposition direction (SS-C) enzymes are involved in the breakdown of sucrose, leading to a reduction in sucrose content. In addition, glucose metabolism is also affected by various factors. The study revealed that varying light qualities have different effects on the activity of sucrose metabolic enzymes in tomato leaves (Li et al., 2017). The application of methyl jasmonate increased the activities of amylase, sucrose phosphate synthase (SPS), and sucrose synthase (SUS) in tomato fruit, meanwhile, it decreased the activities of acid invertase (AI) and neutral invertase (NI). As a consequence, the ripening quality of tomato fruit was enhanced (Li et al., 2022). Water deficit condition positively regulated the expression of sucrose synthase genes, including *SlSUSY4*, *SlSPS1*, *SlCIN3*, *SlVIN2*, and *SlCWIN2*, in tomato fruit, thus promoting the accumulation of sucrose in fruit (Barbosa et al., 2023).

Tomato fruit typically accumulates citric acid and malic acid, along with minor amounts of tartaric acid, oxalic acid, and shikimic acid(Bahrami et al., 2001; Xin et al., 2012). Most of the organic acids in the fruit primarily originate from the TCA cycle. As the main intermediate products in the TCA cycle, their content is affected by the activity of the main metabolic enzymes in the cycle. Phosphoenolpyruvate carboxylase (PEPC) catalyzes the carboxylation of phosphoenolpyruvate (PEP) to produce oxaloacetate acid, and form malic acid under the action of malate dehydrogenase (MDH) (Notton and Blanke, 1993). Then, it is decomposed into pyruvic acid and carbon dioxide under the catalysis of malic enzyme (ME) (Edwards and Andreo, 1992). Moreover, citric acid is generated by citrate synthase (CS) with the participation of acetyl CoA. Under the catalysis of aconitase (ACO), citric acid is initially decomposed to form isocitric acid, and then α-ketoglutaric acid and carbon dioxide are formed under the action of corresponding dehydrogenase (Edwards and Andreo, 1992). During the early stages of tomato fruit ripening, the activity of ME is enhanced, and L-malic acid is decarboxylated to pyruvic acid, and the malic acid content will decrease sharply. Simultaneously, the continuous action of MDH and CS will lead to the reduction of L-malic acid levels, and citric acid will be preferentially accumulated. Therefore, the content of citric acid reaches the maximum at the breaker stage of fruit, and then its content will show different trends (increase, decrease, or flat) with the ripening process, and other acids will continue to decrease during this process (Davies and Maw, 1972; Oms-Oliu et al., 2011). Researchers found that the core metabolic process of grapes (*Vitis vinifera* L.) during ripening is mainly determined by temperature (Rienth et al., 2016). Hot air treatment could significantly up-regulate the degradation of citric acid and fructose and glucose accumulation-related gene expression in ponkan (*Citrus reticulata*) (Chen et al., 2012). In addition to environmental factors, the effects of agronomic measures and exogenous substances on organic acid metabolism cannot be ignored. The treatment with 4% CaCl_2_ treatment exhibited a notable impact on the regulation of malic acid metabolism by promoting the activity of malic acid metabolic enzymes in apples (*Malus pumila* Mill.). This treatment led to the up-regulation of malic acid biosynthesis and transport genes, along with the down-regulation of malic acid degradation genes (Han et al., 2020). Furthermore,1-MCP (1-methylcyclopropene) maintained fruit acidity by modulating the balance between malic acid biosynthesis and degradation (Liu R, et al., 2016).

5-aminolevulinic acid (ALA), a natural and non-toxic plant growth regulator, is a vital precursor for the synthesis of tetrapyrrole compounds in organisms, and it plays a crucial role in regulating plant growth and development (Liu J, et al., 2016). For example, application of exogenous ALA could improve plant tolerance under abiotic stresses (Habiba et al., 2018; Liu et al., 2018; Xiong et al., 2018), enhance photosynthesis(Zhang et al., 2015; Feng et al., 2020), and promote seed germination (WANG et al., 2005; Fu et al., 2014) and fruit quality (Watanabe et al., 2006; Feng et al., 2015). It was found that exogenous ALA treatment enhanced the fixation of peach ^14^C fixation in peach (*Prunus persica* L. Batsch) leaves, promoted the assimilation and transport to fruits, and up-regulate the expression of *SWEET1*, *SWEET6*, *SWEET7* and other genes at fruit ripening stage, and promote fruit sugar accumulation (Ruolin et al., 2023). Rhizosphere application of ALA on apple could improve fruit coloring and increase fruit soluble solids and vitamin C content (Zheng et al., 2017).

Previous study has demonstrated that treating tomato fruits with 200 mg L^-1^ exogenous ALA at the green ripening stage significantly enhances the flavor quality and aromatic quality of the fruits, moreover, this treatment promotes the synthesis and accumulation of lycopene in tomato fruits (Wang J, et al., 2021). Nevertheless, the impacts of exogenous ALA on sugar and acid metabolism in tomato fruits have been scarcely investigated. In this experiment, tomato (*Solanum lycopersicum* cv. Yuanwei No.1) fruits were employed as the test material. Different concentrations of ALA solution were sprayed on the fruits ten days after pollination, and subsequently, the ALA solution was applied once every 10 days until the fruit reached maturity. This research focuses on investigating the regulatory effects of exogenous ALA on the sugar and acid metabolism, as well as the flavor quality of tomato fruit. The study involved measuring the content of soluble sugar, organic acid components, the activity of related metabolic enzymes, and gene expression levels in tomato fruit at different developmental stages. These findings serve as a valuable theoretical foundation for improving high-quality tomato cultivation techniques and the application of ALA in the agricultural domain.

## 2 Material and methods

### 2.1 Plant materials and experimental design

The study employed Tomato (*Solanum lycopersicum* cv. Yuanwei No. 1) obtained from Beijing Lvbaiwang Agricultural Technology Research Institute as the experimental material. The plants were grown and harvested in a solar greenhouse located in Lanzhou City, China (35.87°N, 104.09°E).

After the fourth inflorescence of tomato plants blossomed, the pollination date was recorded on December 17, 2020. Ten days after pollination, the fruits with a diameter of approximately 1 cm were treated with an exogenous ALA solution (0, 100, 200 mg L^-1^). The treatment was administered once every 10 days until the fruit reached maturity, with a total of 5 treatments. Meanwhile, 0.01% Tween-20 was added as a spreading agent in all the sprayed solutions. The concentration of ALA treatment used in this study was based on previous study (Wang J, et al., 2021). Each treatment was replicated three times, with 20 tomato plants randomly selected and marked for each repetition. Tomato fruits were sampled at three different growth stages: the mature green stage (January 8, 2021 to January 15, 2021), the color breaker stage (January 27, 2021 to February 4, 2021), and the maturity stage (February 11, 2021 to February 15, 2021). For each treatment and growth stage, 15 fruits were randomly selected. The fruits were cut into two halves from the central equatorial region. After peeling, the seed and gelatinous placenta were removed. The remaining flesh, columella, and septum tissues were weighed and frozen in liquid nitrogen before being stored at -80°C for later use.

### 2.2 Determination of endogenous ALA content in tomato fruit

The ALA measurement was conducted following the method and adjustment of Wu et al. (Wu Y, et al., 2018). Specifically, 5 g of frozen tomato flesh, columella, and septum samples were placed in a mortar with 6 mL acetate buffer (pH 4.6) in ice bath. After grinding, the sample was transferred into a 10 mL centrifuge tube and centrifuged at 5000 g for 15 minutes at 4°C. Following this, the supernatant (3 mL) was mixed with four drops of acetylacetic ester and incubated at 100°C for 10 minutes in a water bath to initiate a condensation reaction. After cooling to room temperature, a fresh Ehrlich’s reagent solution was prepared by mixing 42 mL of glacial acetic acid, 8 mL of 70% perchloric acid, and 1 g of dimethylaminobenzaldehyde. The resulting mixture was shaken well and placed for color development for 25 minutes. The sample’s absorbance at 554 nm was measured through an ultraviolet spectrophotometer (UV-1800, SHIMADZU, Japan). The ALA content (nmol g^-1^, FW) was determined by utilizing a standard curve of ALA reference standards.

### 2.3 Determination of sugar components in tomato fruit

To extract sugar components, we followed and adjusted the method of Li et al. (Li et al., 2023) subsequently conducted tests on sucrose, fructose, and glucose in tomato fruits using high-performance liquid chromatography (HPLC). We prepared a homogenate of 5 g frozen tomato flesh, columella, and septum tissues using a mortar. The homogenate was then transferred into a 50 mL centrifuge tube (rinsed three times) and diluted to 25 mL. To determine the soluble sugar contents, the fruits were subjected to ultrasonic shaking at 30°C for 60 minutes and then centrifuged at 10,000 rpm for 10 minutes. The resulting supernatant (1.5 mL) was aspirated, and the filtrate was collected through a 0.22 μm filter membrane.

The mobile phase was established as a volume ratio of 3:1 acetonitrile to ultra-pure water. The flowrate was set to 1.0 mL min^-1^ and the column temperature was maintained at 30°C. An injection volume of 10 μL was used and liquid chromatography sugar column LC-NH2 (460 mm×250 mm) was employed. The differential refractive index detector used was Aglient series 1100 (USA). The contents of fructose, glucose, and sucrose were determined in each tissue part of each treatment, which were repeated three times. Total soluble sugar was equal to the sum of fructose, glucose, and sucrose.

### 2.4 Determination of sucrose metabolism-related enzyme activity

To determine the activity of sucrose metabolism-related enzymes in tomato fruits, the following quantitative detection kits were utilized: a plant sucrose synthase (decomposition direction) quantitative detection kit, plant sucrose phosphate synthase (SPS) quantitative detection kit, plant acid invertase (AI) quantitative detection kit, and plant neutral invertase (NI) quantitative detection kit (Ruixin Biotechnology Company Ltd, Quanzhou, China). The specific operation was conducted in accordance with the product description for each kit. In this study, each treatment and tissue were replicated three times. The enzyme activity was calculated based on the standard curve.

### 2.5 Determination of organic acid components in tomato fruit

The levels of shikimate oxalic, malic, citric, quinic, and tartaric acids of tomato fruit were detected using HPLC. The determination method was performed following the chromatographic conditions previously described by Coelho et al. (Coelho et al., 2018) with minor modifications. The frozen tomato fruit tissues (5 g) from the flesh, columella and septum were homogenized using a mortar and then transferred into a 50 mL centrifuge tube (which was rinsed three times) and diluted to 25 mL. Subsequently, the homogenate was centrifuged at 4°C and 10000 rpm for 10 min. After centrifugation, 1.5 ml of supernatant was extracted with a syringe and filtered through a 0.22 μm nylon membrane. A volume of 1.5 mL of the filtered solution was injected for the determination of related components.

For the determination, an HPLC method was employed. The chromatographic column was Aglient C14 (300 mm × 7.7 mm). The detection was conducted using a diode-array detector (DAD) at 210 nm. The mobile phase used was 0.085% phosphoric acid. The column temperature was set to 30°C. The flow rate was 0.8 mL min^-1^, and the injection volume was 20 μL. Each treatment and site were repeated 3 times. The total organic acid content was calculated as the sum of shikimate oxalic, malic, citric, quinic, and tartaric acids.

### 2.6 Determination of citric and malic acid anabolism-related enzyme activity

The activity of enzymes involved in malic acid and citric acid metabolism was determined using quantitative detection kits for phosphoenolpyruvate kinase (PEPC), citrate synthase (CS), aconitase (ACO), malic enzyme (ME), and malate dehydrogenase (MDH) from Ruixin Biotechnology Company Ltd, Quanzhou, China. The specific procedures were conducted in accordance with the product description. Each treatment and tissue was repeated three times. The activity of the relevant was calculated based on the standard curve.

### 2.7 Determination of volatile components content

Gas chromatography-mass spectrometry (GC-MS) was used with reference to the method of Xie et al. (Xie et al., 2022)and slightly modified. The frozen flesh, columella, and septum tissues (3g each) were homogenized and subsequently transferred to a 15 mL brown headspace injection bottle. Chromatographically pure anhydrous Na_2_SO_4_ (1.5 g) and 2-octanol standard sample (10 μL) with a concentration of 88.2 mg L^-1^, were added to the bottle. The mixture was stirred using a magnetic stirring rotor and the cap was quickly tightened after placing the pad. After that, the tomato fruit samples were equilibrated for 10 minutes on a magnetic stirrer set at a constant temperature of 50°C. Subsequently, the extraction needle was inserted into 1/3 of the injection bottle. The extraction process was carried out at 50°C for 30 minutes. Upon completion of the extraction, the extraction needle was inserted into the chromatographic gasification chamber and resolved for 3 minutes before being analyzed using GC-MS for 30 minutes. Each treatment was repeated 3 times.

Only volatile substances with matching degrees > 70 were identified. The content of each volatile aromatic substance was calculated as follows:


$$\text{Content(μg }{\text{kg}}^{-1}\text{)=}\frac{\text{A}1}{\text{A}2}\times \frac{\text{M}1}{\text{M}2}\times 1000$$

Where A1 and A2 are the peak areas of determined and the internal standard, respectively; M1 and M2 are the amount of the internal standard (μg) and sample (g), respectively.

### 2.8 Total RNA extraction and relative gene expression analysis

Total RNA was extracted using the RNAprep Pure Plant Plus Kit (Tiangen Biotech, Beijing, China) according to the manufacturer’s protocol. The cDNA synthesis was performed using a FastKing RT Kit (Tiangen Biotech) according to the manufacturer’s protocol. The tomato actin gene was used as an internal control. Quantitative analysis was performed using the Light Cycle 96 RT-qPCR instrument (Roche, Switzerland). The GenBank accession numbers of the sequences used to design the primers are listed in **Table 1**. The relative expression of genes was calculated by the 2^-ΔΔCT^ method, and each gene expression analysis was performed in three independent biological replicates.

**Table 1:** Primer sequences used for quantitative RT-qPCR.

Gene	Accession number	Forward primer 5’–3’	Reverse primer 5’–3’
*SlAI*	NM_001247914.2	CTCCGCCTCTCGTTACACATTACTC	CGGTGACTGGTTGTTGAGGATCG
*SlNI*	XM_004250984.4	TTCCGACTTTGTTGGTGACTGATGG	AGGTCTGTTGATGCCTCCTCTGG
*SlCS*	XM_004251765.2	CCACCTGTCCTTCCATCCAACAATC	AGAATATCGAGCACACGAGCAAGTC
*SlSS*	MG962510.1	GGTACGCCAAGAATCCACGACTAAG	CTTCTTCATCTCTGCCTGCTCTTCC
*SlSPS*	NM_001246991.2	TGGTCTACGCAAGGCTGTCATAATG	CTGCTACATTCCTCGTCTGCTTGG
*SlPPC2*	NM_001320894.1	GAAAGGATGCTGGTCGGCTGTC	TCCTCTTCCAACTGTACCACCTCTG
*SlME1*	NM_001247529.2	GCCCTCTCCAATCCAACATCACAG	CCAGACGCATAGACCTTCCCATTG
*SlmMDH1*	NM_001247072.2	TGTTGCCGCCGATGTTAGTCAC	GCACACCAGCAGGAATGATAACAAC
*SlACO*	XM_004243424.4	TGCTTCAACTTCTACTGCTGCTCAC	AGACTTCCAATCAACACCGTGACTC
*Actin*	NM_001330119.1	ATTGTGTTGGACTCTGGTGATGGTG	GACGGAGAATGGCATGTGGAAGG

### 2.9 Statistical analysis

Statistical analysis and data visualization were performed using Excel 2016 and Origin 2022 software (OriginLab, Northampton, MA, USA). For data analysis, SPSS version 23.0 (IBM Corp., Armonk, NY, United States) was used. Duncan’s multiple range tests of variance (*P*< 0.05) were employed for significance testing.

## 3 Results

### 3.1 The endogenous ALA changes during fruit development

**Figure 1:**
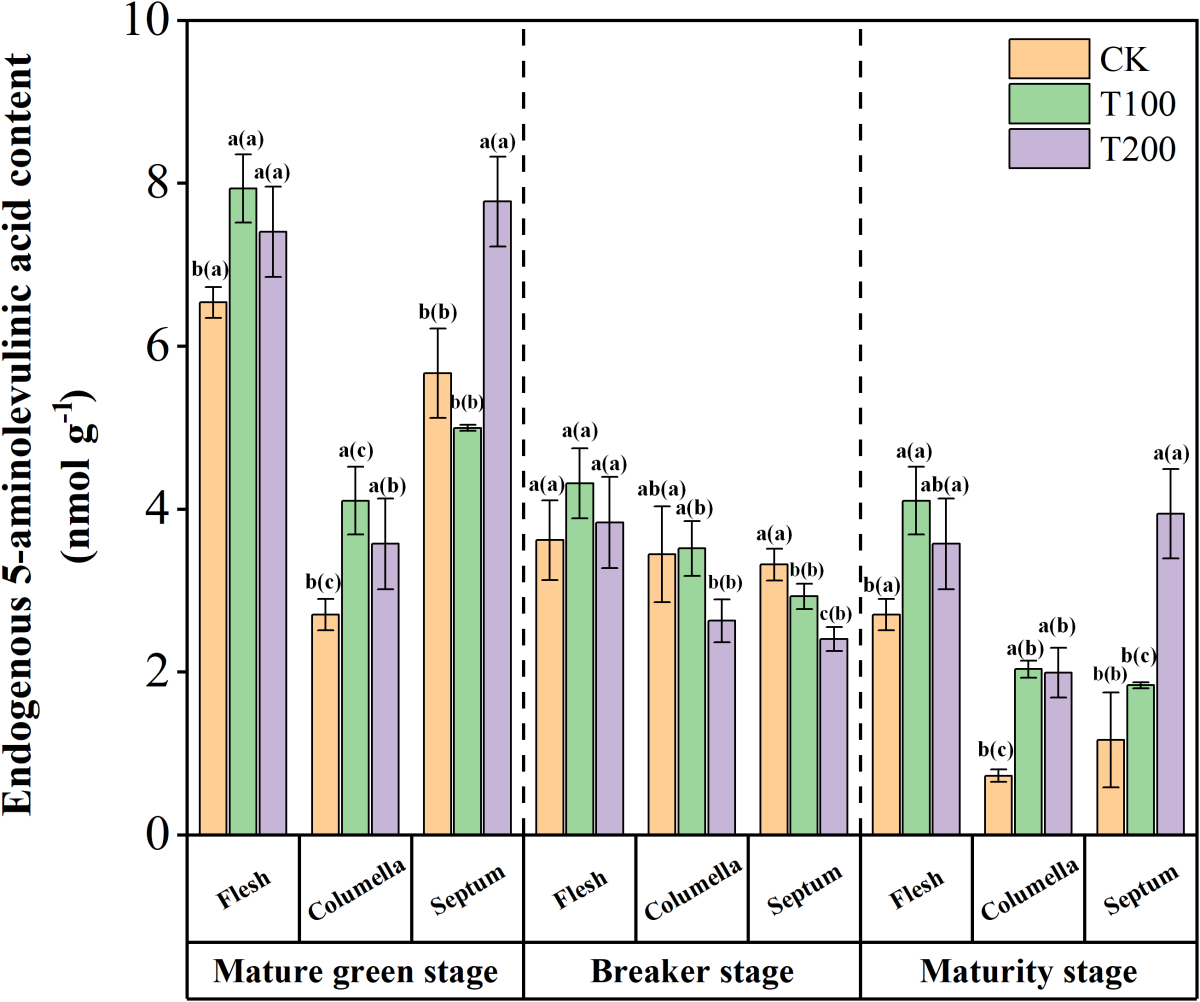
Effects of exogenous ALA on endogenous ALA content in tomato fruit. The short vertical line in the histogram represents the mean ± SE (n=3), and the significance level is *P*< 0.05. The letters in brackets indicate the difference level between different fruit tissues under the same treatment. The letters outside the brackets indicate the difference level between different treatments in the same fruit tissue.

Endogenous ALA was identified as the precursor of all tetrapyrrole compounds found in tomato fruit, as illustrated in **Figure 1**. At the mature green stage, the flesh and septum of the tomato fruit exhibited higher ALA content compared to the columella. After treated with exogenous ALA, the endogenous ALA content in each tissue increased, with a significant increase observed in the T200 treatment. However, at the breaker stage, the endogenous ALA content in the flesh and septum decreased rapidly. Moreover, the endogenous ALA content in the septum tissue was significantly reduced under T100 and T200 treatments, with a reduction of 11.7% and 27.5% respectively compared to the control. Additionally, at the maturity stage, the endogenous ALA content decreased rapidly in the columella and septum.

### 3.2 The total sugar and organic acid changes during fruit development

**Figure 2:**
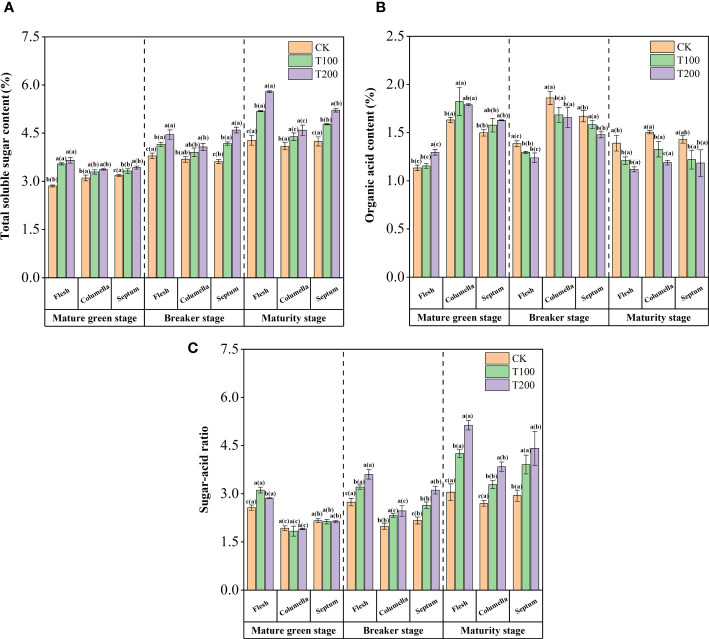
Effect of exogenous ALA on the tomato content of: total soluble sugar **(A)**, total organic acid **(B)**and sugar-acid ratio **(C)**. The short vertical line in the histogram represents the mean ± SE (n=3), and the significance level is *P*< 0.05. The letters in brackets indicate the difference level between different fruit tissues under the same treatment. The letters outside the brackets indicate the difference level between different treatments in the same fruit tissue.

The flesh of T100 and T200 treatments revealed a significant increase in total soluble sugar content by 22.0% and 35.8% respectively, compared to the control (**Figure 2A**). In comparison to the control group, the total soluble sugar content in the columella increased significantly by 8.3% and 12.9%, respectively. Similarly, the septum exhibited a significant increase in total soluble sugar content by 11.5% and 23.3%. The soluble sugar content in the fruit treated with exogenous ALA was found to be the highest in the flesh, followed by septum and columella, with significant differences observed among the different tissues. It indicated that exogenous ALA not only promoted the synthesis and accumulation of sugars, but also facilitated their translocation to the flesh. Notably, the T200 treatment exhibited the most pronounced effect.

The ALA treatments at the mature green stage resulted in an increase of organic acids content in fruits (**Figure 2B**). However, from the beginning of the breaker stage to the maturity stage, the content of organic acids in different tissues of the tomato fruits significantly decreased. In tomato fruits, organic acids were predominantly accumulated in the columella. However, exogenous ALA treatments resulted in a significant reduction in the total organic acid content in the columella, with the T200 treatment exhibiting the most significant effect.

In addition, exogenous ALA treatments could significantly increase the sugar-acid ratio in various tissues of tomato fruit at different stages (**Figure 2C**). At the mature green stage, the sugar-acid ratio in the flesh exhibited a significant increase due to ALA treatments. Moreover, during the breaker and maturity stages, the sugar-acid ratio in the tissues of fruits treated with T100 and T200 showed a significant increase compared to the control group. In addition, the variation of sugar-acid ratio in each tissue of every treatment demonstrated a similar pattern, with the highest ratio observed in the flesh, followed by the septum, and the lowest in the columella. Notably, the T200 treatment had the most profound impact on the changes in the sugar-acid ratio.

### 3.3 Sugar components were enhanced by exogenous ALA during fruit development

**Figure 3:**
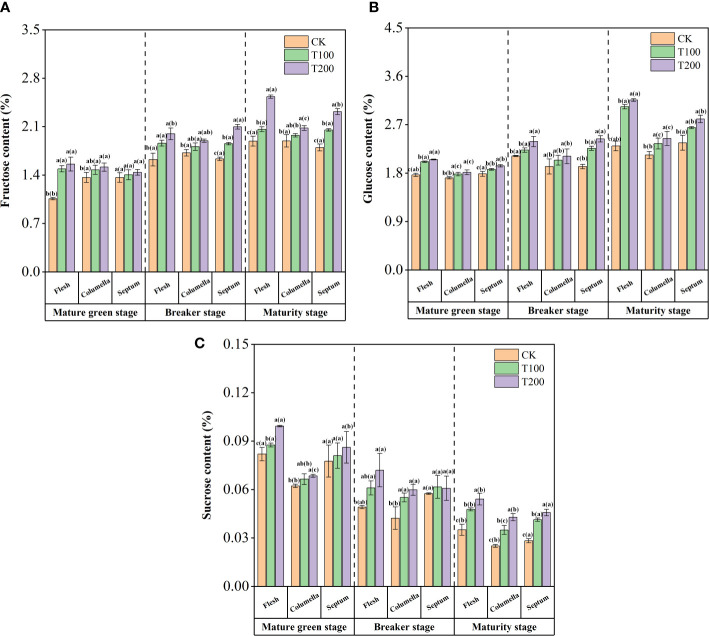
Effect of exogenous ALA on the tomato content of: fructose **(A)**, glucose **(B)** and sucrose **(C)**. The short vertical line in the histogram represents the mean ± SE (n=3), and the significance level is *P*< 0.05. The letters in brackets indicate the difference level between different fruit tissues under the same treatment. The letters outside the brackets indicate the difference level between different treatments in the same fruit tissue.

The contents of fructose, glucose and sucrose were increased under the application of exogenous ALA in tomato fruits (**Figure 3**). During fruit development, the application of exogenous ALA resulted in a significant increase in fructose content in various tissues (**Figure 3A**). At the maturity stage, the fructose content in the flesh of T100 and T200 treatments was significantly increased by 9.1% and 34.0%, respectively, compared with the control fruit. The fructose content in the septum was significantly increased by 14.2% and 28.8%, respectively, while the fructose content in the columella was significantly increased by 8.14% and 13.3%, respectively, in comparison to the control fruit. The fructose content in the flesh of the T100 treated fruit was significantly higher than that of the columella and the septum. The fructose content of T200 treatment found to be highest in the flesh followed by the septum and the columella. These findings indicate that exogenous ALA not only significantly increases the overall fructose content of the fruit but also promotes preferential fructose accumulation in the flesh. Notably, the T200 treatment exhibited the most pronounced effect in this regard.

During the ripening process of tomato fruit, the glucose content showed an increasing trend (**Figure 3B**). At the mature green stage, T100 and T200 treatments significantly increased the glucose content in the flesh, which increased by 15.4% and 16.1%, respectively, compared with the control. Under the same treatment conditions, the content in the columella was the lowest, followed by the septum. At the breaker stage, ALA treatments significantly increased the glucose content in each tissue. At the maturity stage, the glucose content in the flesh of T100 and T200 treated fruits was significantly higher than that of the control. This promotive effect was similarly observed in columella and septum. The fruit treated with T100 and T200 exhibited the highest glucose content in the flesh, followed by the septum and columella. Notably, there were significant differences in glucose content between each tissue.

During the mature green stage, the content of sucrose was the highest in the fruit flesh, followed by septum, and then columella. The breaker and maturity stages showed the highest in the septum, followed by the flesh, and the least in the columella. The sucrose accumulation in different tissues of ALA-treated tomato fruit increased differently. At the mature green stage, both T100 and T200 treatments led to significant increases in sucrose content across different tomato tissues. Specifically, sucrose content increased by 6.9% and 21.1% in the flesh, 6.7% and 10.0% in the columella, and 4.4% and 11.0% in the septum, respectively. During the breaker period, the sucrose content in flesh and columella increased significantly. At the maturity stage, ALA treatments significantly increased the sucrose content in each tissue. In T200 treatment, the sucrose content in flesh, columella and septum increased by 61.9%, 71.1% and 54.5% compared with the control group, and T100 treatment increased by 46.2%, 39.4% and 36.2%, respectively (**Figure 3C**).

### 3.4 The sucrose metabolic enzymes and gene expression during fruit development

**Figure 4:**
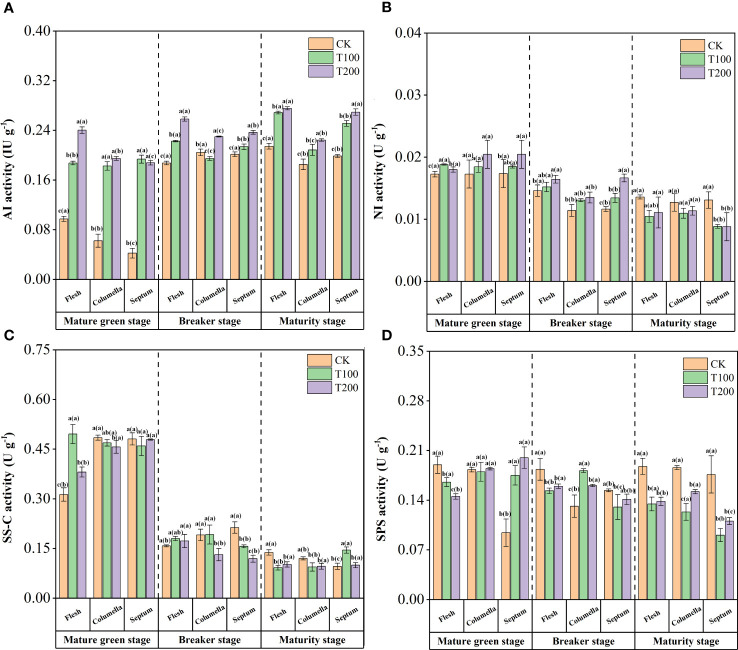
Effects of exogenous ALA on the activities of sucrose metabolic enzymes in different developmental stages and tissues of tomato fruits. **(A)** AI, **(B)** NI, **(C)** SS-C and **(D)** SPS. The short vertical line in the histogram represents the mean ± SE (n=3), and the significance level is *P*< 0.05. The letters in brackets indicate the difference level between different fruit tissues under the same treatment. The letters outside the brackets indicate the difference level between different treatments in the same fruit tissue.

ALA treatments remarkably increased the AI activity of each tissue in each stage (**Figure 4A**). At the mature green stage, the activity of AI increased most obviously, reaching 0.6 to 2.5 times compared to that of control. Throughout the breaker stage and the maturity stage, the most substantial increase in AI activity within ALA treatments was observed in the flesh and septum tissues.

The ALA treatments enhanced NI activity at the mature green stage (**Figure 4B**). T200 treatment significantly increased the NI activity by 4.3% and 17.9% in flesh and septum, respectively. At the breaker stage, the NI activity in the flesh was significantly higher than that in the other tissues. T100 and T200 treatments significantly increased by 4.1% and 12.5% in flesh, 14.6% and 18.4% in the columella, and 15.5% and 43.2% in the septum. At the maturity stage, the activity of NI in each tissue decreased after ALA treatments, and the activity in the flesh was higher, followed by the columella, and the septum was the lowest.

The activity of sucrose synthase (decomposition) (SS-C) gradually decreased with fruit ripening (**Figure 4C**). At mature green stage, exogenous ALA significantly increased SS-C activity in flesh, but decreased it in columella, with the T200 treatment exhibiting a reduction of 5.8% compared to the control. During the breaker stage, the SS-C activity in septum of T100 and T200 treatments was significantly reduced by 26.6% and 44.2% compared to that of control. At the maturity stage, the SS-C activity of each tissue was significantly reduced.

The SPS activity was the lowest in the septum at the mature green stage (**Figure 4D**). ALA treatments could significantly increase the activity of SPS in the septum. At the breaker stage, ALA treatments significantly reduced the SPS activity in the flesh and septum, but the opposite in the columella. At the maturity stage, the SPS activity in flesh, columella and septum of ALA-treated fruits decreased significantly, among which T100 treatment decreased by 28.2%, 33.5% and 48.5%, and T200 treatment decreased by 26.2%, 18.2% and 37.4%.

**Figure 5:**
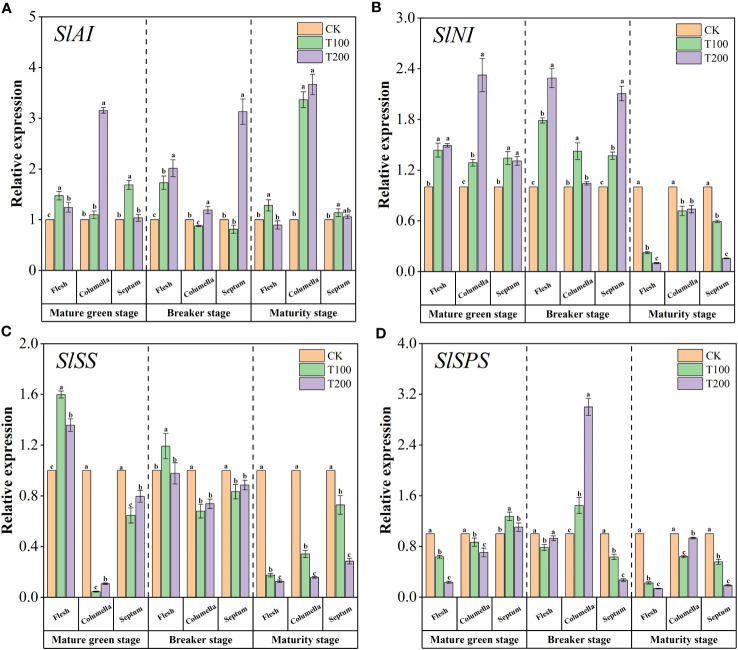
Effects of exogenous ALA on the relative expression of key enzyme genes in sucrose metabolic enzymes in different developmental stages and tissues of tomato fruits. **(A)** *SlAI*, **(B)** *SlNI*, **(C)** *SlSS* and **(D)** *SlSPS*. The short vertical line in the histogram represents the mean ± SE (n=3), and the significance level is *P*< 0.05. Different lowercase letters indicate the difference level between different treatments in the same fruit tissue.

The relative expression levels of the four key genes involved in the glucose metabolism pathway were shown in the **Figure 5**. ALA treatments increased the expression level of *SlAI* (**Figure 5A**). Among them, the expression level of *SlAI* in the columella T200 treated at the mature green stage was 3.15 times that of the control. The expression of *SlAI* in the columella treated with T100 and T200 were 3.37 and 3.67 times higher that than of control, respectively. The expression levels of *SlNI* in flesh, columella and septum were significantly up-regulated under ALA treatments at mature green stage and breaker stage (**Figure 5B**). Besides, at the mature green stage, ALA treatments significantly increased the expression level of *SlSS* in the flesh, conversely, the opposite effect was observed in both the columella and the septum (**Figure 5C**). During the breaker stage, T100 treatment significantly up-regulated the expression of *SlSS* in the flesh. During maturity stage, ALA treatments significantly down-regulated the expression of *SlSS* in various tissues of fruit. As shown in the **Figure 5D**, at the mature green stage, ALA treatments significantly down-regulated the expression of *SlSPS* in flesh and columella. Compared with the control, T100 treatment significantly up-regulated the expression of *SlSPS* in the septum. At the breaker stage, ALA treatments only up-regulated the expression of *SlSPS* in the columella, and the expression of *SlSPS* in the columella treated with T100 and T200 was 1.44 and 3 times higher than that of the control. At maturity stage, ALA treatments significantly down-regulated the expression of *SlSPS* in various fruit tissues.

### 3.5 Organic acids were regulated by exogenous ALA during fruit development

**Figure 6:**
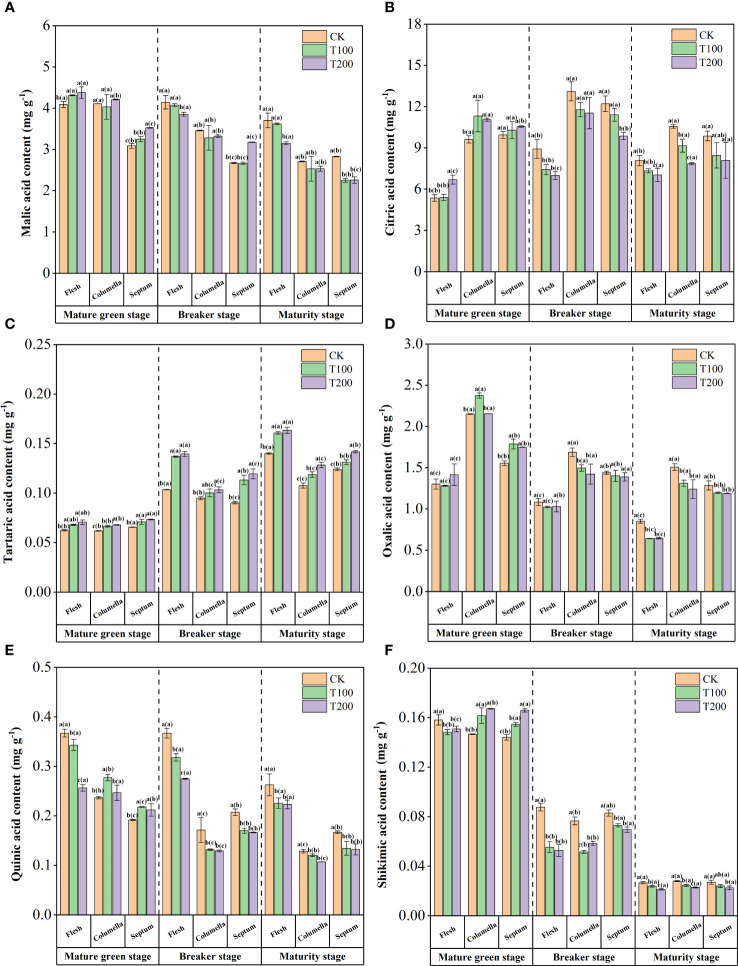
Effects of exogenous ALA on the content of malic acid **(A)**, citric acid **(B)**, shikimic acid **(C)**, quinic acid **(D)**, tartaric acid **(E)** and oxalic acid **(F)**. The short vertical line in the histogram represents the mean ± SE (n=3), and the significance level is *P*< 0.05. The letters in brackets indicate the difference level between different fruit tissues under the same treatment. The letters outside the brackets indicate the difference level between different treatments in the same fruit tissue.

The content of malic acid decreased gradually during the ripening process of tomato fruit (**Figure 6A**). The content of malic acid in the flesh was the highest, followed by the columella and the least septum. During the mature green stage, ALA treatments significantly increased the malic acid content in flesh and septum. T100 treatment was 5.4% and 5.2% higher than that of the control treatment, and T200 treatment was 7.0% and 14.1% higher than that of the control treatment. During the breaker stage and maturity stage, the effect of malic acid content in the flesh was opposite to that of the mature green stage. The effects of exogenous ALA were mainly concentrated in the flesh and septum.

During fruit development, the content of citric acid reached the highest at the breaker stage, showing a trend of increasing first and then decreasing (**Figure 6B**). At the mature green stage, ALA treatments significantly increased the citric acid content in the columella of tomato fruit. During the breaker stage, ALA treatments showed the effect of reducing the content. During the maturity stage, citric acid was reduced, and ALA treatments could promote this reduction, still maintaining the content gradient of columella > septum > flesh. T200 treatment effect was most obvious, significantly reduced the citric acid content in the flesh and the columella, which was significantly lower than the control by 12.9% and 25.6%, respectively.

Furthermore, there was a higher accumulation of tartaric acid and quinic acid in the flesh, while oxalic acid exhibited higher accumulation in the columella, and shikimic acid showed a greater accumulation in the septum. In addition, the application of exogenous ALA during fruit development could reduce the content of acids in various tissues, but the content of tartaric acid showed an increasing trend (**Figure 6C**).

During the process of fruit ripening, the content of oxalic acid was highest in the columella tissue, followed by the septum, and lowest in the flesh (**Figure 6D**). The ALA treatments had no effect on the oxalic acid content in the flesh tissue, but increased it in other tissues at the mature green stage of fruit. During the breaker stage, T100 and T200 treatments significantly reduced the oxalic acid content in the columella, which was 11.2% and 15.7% lower than that of the control. At the maturity stage, the oxalic acid content in each tissue under ALA treatments was significantly decreased.

ALA treatments significantly reduced the content of quinic acid in flesh tissue in three stages (**Figure 6E**). The content of quinic acid increased in columella and septum tissues under ALA treatments at mature green stage.

Moreover, ALA treatments reduced the content of shikimic acid in the flesh tissue at the mature green stage. Specifically, the T100 and T200 treatments led to decrease of 6.36% and 5.1%, respectively, compared with the control. Contrarily, ALA treatments increased the content of shikimic acid in columella and septum. The content of shikimic acid in each tissue was significantly reduced at the breaker stage under ALA treatment, especially in the flesh and the columella. The ALA treatments could significantly reduce the content of shikimic acid in maturity stage, and the effect of T200 ALA treatment was the most significant (**Figure 6F**).

### 3.6 The main organic acids metabolic enzymes and gene expression during fruit development

**Figure 7:**
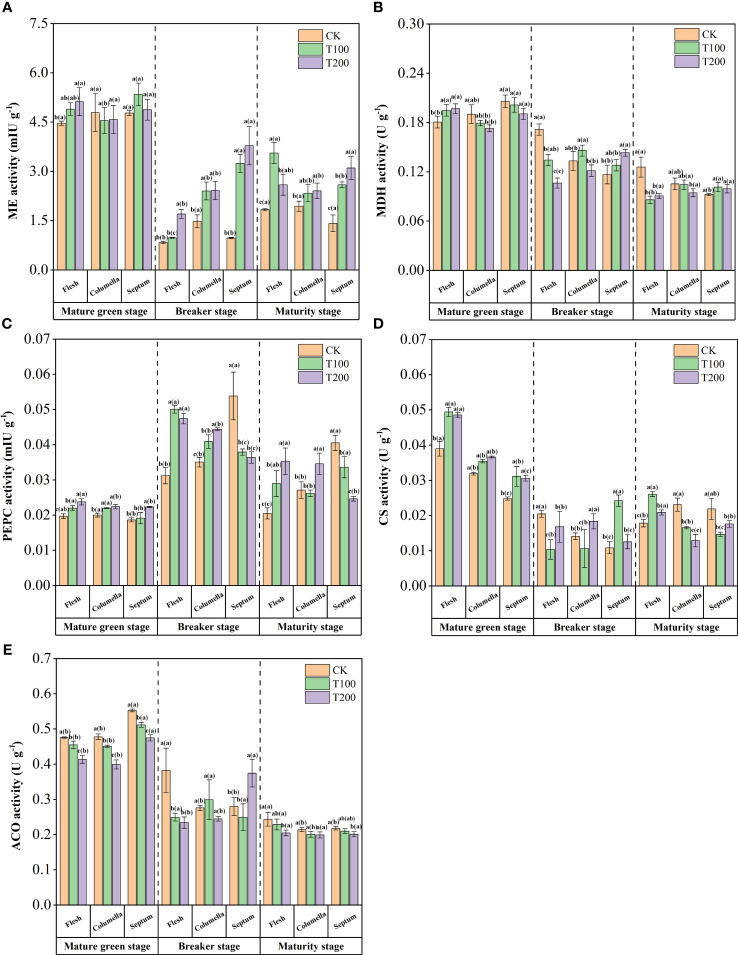
Effects of exogenous ALA on the activities of main organic acids metabolic enzymes in different developmental stages and tissues of tomato fruits. **(A)** ME, **(B)** MDH, **(C)** PEPC, **(D)** CS and **(E)** ACO. The short vertical line in the histogram represents the mean ± SE (n=3), and the significance level is *P*< 0.05. The letters in brackets indicate the difference level between different fruit tissues under the same treatment. The letters outside the brackets indicate the difference level between different treatments in the same fruit tissue.

At the mature green stage, ME activity in flesh tissue was significantly increased under ALA treatment compared with the control. The ME activity decreased significantly at the beginning of the breaker stage, and ALA treatments could significantly increase the ME activity at this stage, with the most prominent effect of T200 treatment. At the maturity stage, there were differences between the various tissues under the T100 treatment, and the flesh was 34.3% and 18.0% higher than the columella and the septum. High ME activity resulted in the decrease of malic acid content in ALA treatments at the breaker stage and maturity stage (**Figure 7A**).

MDH activity varied during fruit development after ALA treatments (**Figure 7B**). At the mature green stage, the MDH activity in the flesh of T100 and T200 treatments increased by 7.9% and 9.0% compared with the control, while the opposite changes were observed in the columella. During the fruit breaker stage, ALA treatments significantly reduced the MDH activity in the flesh, which was 21.6% (T100) and 38.0% (T200) lower than that of the control, respectively. During the maturity stage, ALA treatments also significantly reduced the MDH activity in the flesh. These results indicated that exogenous ALA could reduce MDH activity, reduce the content of malic acid, and have a greater effect on flesh tissue.

At mature green stage, T200 treatment significantly increased PEPC activity in flesh, columella and septum, which was 20.6%, 12.0% and 19.8% higher than that of the control. During the breaker stage, ALA treatments continued to increase the PEPC activity in the flesh and columella. In contrast, ALA treatments significantly decreased PEPC activity in septum. At the maturity stage, higher PEPC activity was formed in the flesh and columella under ALA treatment, and the septum was lower (**Figure 7C**).

The activity of CS decreased at breaker stage and increased again at maturity stage in fruit (**Figure 7D**). At mature green stage, T100 and T200 treatments significantly increased CS activity by 26.8% and 24.6% in flesh, by 11.0% and 14.8% in columella, and by 25.5% and 23.3% in septum. At the maturity stage, ALA treatments increased CS activity in flesh, and exerted a significant impact on CS activity in both columella and septum. Notably, CS activity exhibited a high expression level in the flesh.

ACO activity gradually decreased with fruit ripening, and had the high activity at the mature green stage (**Figure 7E**). At this stage, there was a high-throughput citric acid synthesis and transformation process. ALA treatments inhibited ACO activity at mature green stage. At maturity stage, ACO activity decreased significantly under T200 treatment in flesh and septum, and there was no change in columella, which resulted in the decrease of citric acid content at maturity stage.

**Figure 8:**
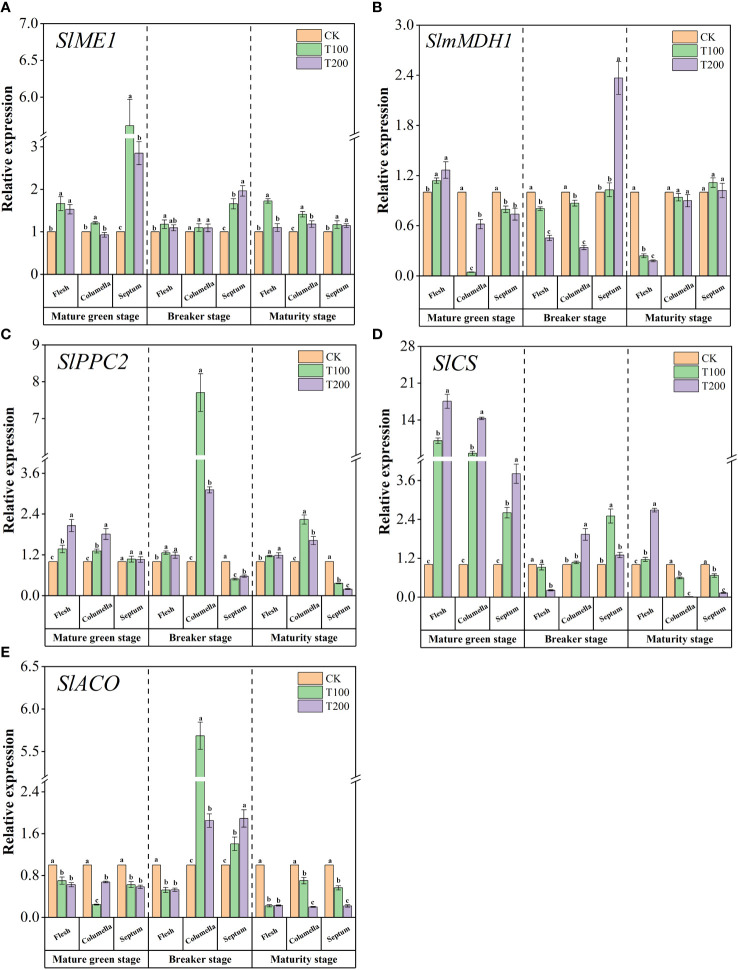
Effects of exogenous ALA on the relative expression of key enzyme genes in main organic acids metabolic enzymes in different developmental stages and tissues of tomato fruits. **(A)** *SlME1*, **(B)** *SlmMDH1*, **(C)** *SlPPC2*, **(D)** *SlCS* and **(E)** *SlACO*. The short vertical line in the histogram represents the mean ± SE (n=3), and the significance level is *P*< 0.05. Different lowercase letters indicate the difference level between different treatments in the same fruit tissue.

We studied the relative expression levels of five key genes involved in the acid metabolism pathway (**Figure 8**). At the mature green stage, compared with the control fruit, ALA treatments significantly up-regulated the expression of *SlME1* in various tissues of the fruit (**Figure 8A**). During the breaker stage, ALA treatments increased the expression level of *SlME1* in the septum. At the maturity stage, T100 treatment significantly increased the expression of *SlME1* in the flesh, columella and septum, which were 2.72, 2.41 and 2.17 times higher than those of the control, respectively. As shown in the **Figure 8B**, at the mature green stage, ALA treatments significantly increased the expression level of *SlmMDH1* in the flesh and decreased the expression level of *SlmMDH1* in the columella and septum. During the breaker period, ALA treatments significantly down-regulated the expression of *SlmMDH1* in flesh and columella, and T200 treatment significantly increased the expression of *SlmMDH1* in septum, which was 2.37 times higher than that of the control. At the maturity stage, ALA treatments significantly down-regulated the expression of *SlmMDH1* in the flesh. Besides, ALA treatments increased the expression level of *SlPPC2* in each stage flesh and septum, and decreased the expression level of *SlPPC2* in the septum at the breaker and maturity stages (**Figure 8C**). In addition, the application of ALA notably up-regulated the expression of *SlCS* in various fruit tissues during the mature green stage. At maturity stage, T200 treatment significantly up-regulated the expression of *SlCS* in flesh (**Figure 8D**). ALA treatments significantly down-regulated the expression of *SlACO* in various tissues at the mature green stage and the maturity stage. At the breaker stage, ALA treatments significantly increased the expression level of *SlACO* in the columella and septum (**Figure 8E**).

### 3.7 Volatile quality of tomato fruit was promoted by exogenous ALA

**Figure 9:**
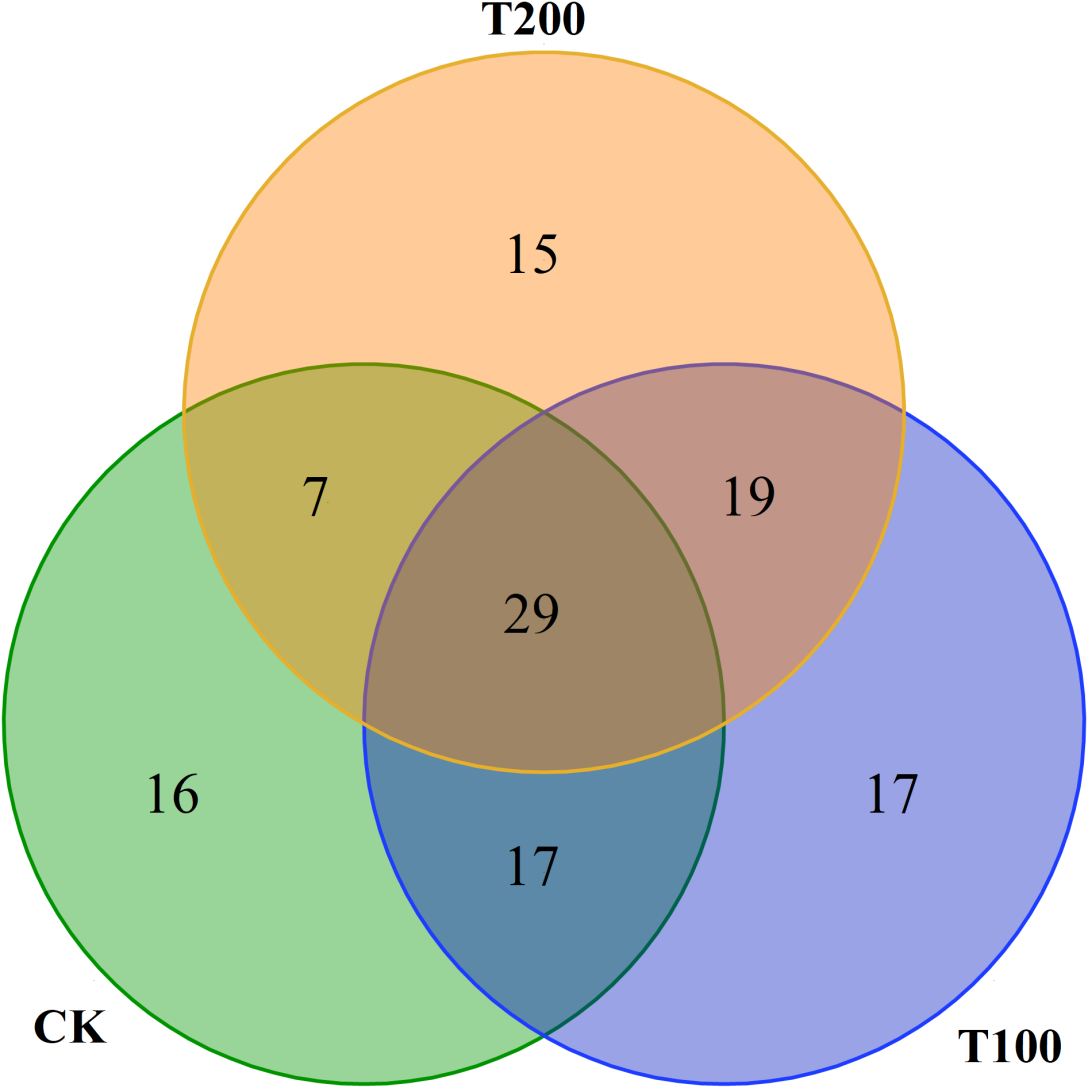
The Venn diagram in effect of exogenous ALA on amount of volatile compounds in tomato fruit at maturity stage. Each circle represents each treatment, the intersection of the circle and the circle represents the common volatile substances between different treatments, and the number of intersecting parts represents the number of common volatile substances.

In this study we identified a total of 114 distinct volatiles in mature tomato fruits (**Table 2**). These volatiles were categorized into seven groups: hydrocarbons (7 to 19 types), aldehydes (17 to 23 types), alcohols (11 types), esters (5 types), ketones (10 to 11 types), phenols (5 to 8 types), and other substances (3 to 8 types). The application of exogenous ALA treatments resulted in a significant increase in both the total number and mass fraction of volatile substances. The number of CK species was 69, while T100 and T200 treatments had 81 and 68 species respectively. Regarding the total mass fraction, T100 (5489.75 μg kg^-1^) and T200 (4398.39 μg kg^-1^) showed higher values than CK (3232.47 μg kg^-1^), with T100 being 0.7 times and T200 being 0.36 times of CK. The highest content in ALA treatments was trans-2-hexenal, which was not detected in CK treatment. The highest content in CK treatment was 2-cyclohexen-1-ol, which was also not detected in ALA treatments. There were 29 common volatile compounds detected in the three treatments, accounting for 24.58% of the total (**Figure 9**).

**Table 2:** Effects of exogenous ALA on the categories and contents of volatile compounds in tomato fruit at maturity stage.

Categories	Volatile compounds	Chemical formula	Retention time/min	Content (µg kg^-1^)		
				CK	T100	T200
Hydrocarbons	(E)-4,5-Epoxy-(E)-2-decenal	C_10_H_16_O_2_	34.61	24.29	37.63	27.61
	1,2-Dihydrothujopsene-(I1)	C_15_H_26_	24.60	19.12	–	–
	α-Copaene	C_15_H_24_	18.53	36.34	26.14	–
	Styrene	C_8_H_8_	10.57	11.14	19.27	–
	p-Xylene	C_8_H_10_	6.89	8.02	–	–
	3-methyl-1-Octene	C_9_H_18_	10.71	6.19	8.27	–
	Dodecane	C_12_H_26_	8.33	26.96	–	–
	Tridecane	C_13_H_28_	11.96	6.72	4.38	–
	1-Octadecyne	C_18_H_34_	17.31	0.82	5.08	–
	1-isopropyl-4,5-dimethyl-Cyclopentene	C_10_H_18_	19.98	6.10	7.29	–
	1-Phenyl-1-butene	C_10_H_12_	16.72	7.97	–	–
	p-Cymene	C_10_H_14_	10.97	7.93	8.84	–
	1-Tetradecyne	C_9_H_14_O	18.12	–	1.35	–
	o-Xylene	C_8_H_10_	6.54	–	5.72	–
	1-methyl-4-(1-methylethyl)-1,3-Cyclohexadiene	C_10_H_16_	25.58	–	44.48	–
	1-propyl-Cyclohexene	C_10_H_18_O	15.52	–	98.27	–
	(1S-(1α,3β,6α))-3,7,7-Trimethyl-bicyclo[4.1.0]heptane	C_10_H_18_	15.13	-	8.45	6.48
	.alpha.-Cubebene	C_15_H_24_	17.45	43.12	45.01	19.19
	Cis-bicyclo[4.3.0]nonane	C_9_H_16_	15.56	–	82.31	47.66
	1-ethenyl-3,5-dimethyl-Benzene	C_10_H_12_	15.75	–	7.28	5.03
	Germacrene D	C_15_H_24_	17.61	–	89.89	–
	Orcinol	C_13_H_22_O	33.83	–	19.78	–
	(1-methylbutyl)-Oxirane	C_7_H_14_O	14.38	–	4.04	–
	Naphthalene	C_10_H_8_	26.13	–	–	5.30
Ketons	3,5-dihydroxy-2-methyl-4H-Pyran-4-one	C_8_H_14_O	42.15	–	52.97	48.18
	6-methyl-5-Hepten-2-one	C_8_H_14_O	13.41	121.53	149.30	188.57
	5-methyl-4-Hexen-3-one	C_7_H_12_O	22.16	31.56	26.88	14.08
	1-Hepten-3-one	C_7_H_12_O	11.71	34.46	11.74	–
	1-Penten-3-one	C_5_H_8_O	4.34	34.84	–	–
	4-(2,2,6-trimethyl-7-oxabicyclo[4.1.0]hept-1-yl)-3-Buten-2-one	C_13_H_20_O_2_	34.18	23.23	37.63	15.08
	(E)-6,10-Dimethyl-5,9-undecadien-2-one	C_13_H_22_O	30.39	91.01	92.69	54.88
	β-Ionone	C_13_H_20_O	32.72	45.64	68.40	48.56
	4-(2,6,6-trimethyl-2-cyclohexen-1-yl)-3-buten-2-one	C_13_H_20_O	30.22	16.73	–	–
	3,3,6-trimethylhepta-1,5-dien-4-one	C_10_H_16_O	6.78	48.95	–	–
	5-(1-methylethyl)-bicyclo[3.1.0]hexan-2-one	C_9_H_14_O	15.58	94.45	–	–
	1-(2,6,6-Trimethyl-1,3-cyclohexadien-1-yl)-2-buten-1-one	C_13_H_18_O	29.12	13.00	12.85	19.02
	2,3-Dihydro-3,5-dihydroxy-6-methyl-4H-pyran-4-one	C_11_H_10_O_6_	41.61	–	168.50	164.73
	1-(3-Hydroxyphenyl)-ethanone	C_8_H_8_O_2_	28.54	–	–	20.40
	4’-Hydroxy-acetophenone	C_8_H_8_O_2_	28.53	–	17.37	16.69
Phenols	(Z)-2-Methoxy-4-(1-propenyl)-phenol	C_10_H_12_O_2_	43.54	1.33	29.96	–
	4-Propyl-phenol	C_9_H_12_O	12.43	–	13.79	34.68
	Cis-decahydro-1-naphthol	C_10_H_18_O	11.27	–	8.17	–
	3-(1-Methylethyl)-phenol	C_9_H_12_O	37.75	3.93	–	–
	2-Propyl-phenol	C_9_H_12_O	13.22	7.42	8.59	12.75
	2-Ethyl-phenol	C_8_H_10_O	36.60	7.74	–	–
	2-Methoxy-phenol	C_7_H_8_O_2_	30.48	138.06	–	58.41
	4-(1-Methylpropyl)-phenol	C_10_H_14_O	29.89	25.22	52.08	14.40
	3,5-Dimethyl-phenol	C_8_H_10_O	10.35	8.33	–	–
	Eugenol	C_10_H_12_O_2_	39.00	16.18	33.25	60.28
Alcohols	10-Methyltricyclo[4.3.1.1(2,5)]undecan-10-ol	C_12_H_20_O	27.02	6.14	12.75	12.35
	(S)-Cis-verbenol	C_10_H_16_O	24.97	16.11	30.37	12.51
	Trans-Verbenol	C_7_H_8_O_2_	24.56	–	13.91	29.65
	Cis-2-methylcyclohexanol	C_7_H_14_O	15.44	25.42	–	–
	Cis-2-methylcyclopentanol	C_6_H_12_O	5.63	102.20	304.99	–
	(E)-2-methylcyclopentanol	C_6_H_12_O	7.63	–	238.24	–
	1-Octen-3-ol	C_8_H_16_O	17.55	12.71	15.30	11.50
	(+/-)-1,2-Diphenyl-1,2-ethanediol	C_14_H_14_O_2_	31.06	14.40	25.75	–
	Linalool	C_10_H_18_O	20.85	29.96	20.87	23.82
	2-Cyclohexen-1-ol	C_6_H_10_O	9.59	1352.91	–	–
	alpha-Terpineol	C_10_H_18_O	25.65	30.84	5.26	16.50
	3-Furanmethanol	C_6_H_12_O_2_	24.59	–	46.35	–
	3-Methylhex-5-en-3-ol	C^6^H^10^O	14.88	–	26.88	–
	(E)-3-Methylpenta-1,3-diene-5-ol	C_6_H_10_O	8.81	64.36	–	25.28
	Benzyl alcohol	C_7_H_8_O	31.08	–	–	15.12
	3-Cyclopentyl-1-propanol	C_8_H_16_O	15.46	–	–	46.10
	1-(1-Butyny) cyclopentanol	C_5_H_10_O	18.46	–	–	11.48
	(E)-4-Hexen-1-ol	C_6_H_12_O	15.46	46.42	–	53.97
Aldehydes	Heptanal	C_7_H_14_O	8.30	10.52	2.14	–
	Decanal	C_10_H_20_O	18.97	28.30	1.49	–
	Octanal	C_8_H_16_O	11.68	11.64	8.03	3.50
	Nonanal	C_9_H_18_O	15.30	41.59	39.06	7.06
	(E)-2-Heptenal	C_7_H_12_O	12.87	93.30	117.39	48.73
	(E)-2-Nonenal	C_9_H_16_O	20.17	28.27	–	–
	(E,E)-2,4-Decadienal	C_10_H_16_O	27.61	14.93	18.10	12.46
	(E)-2-Decenal	C_10_H_18_O	23.76	25.13	32.07	21.30
	1,3,4-Trimethyl-3-cyclohexene-1-carboxaldehyde	C_10_H_16_O	25.95	2.10	–	–
	(E,E)-2,4-Heptadienal	C_7_H_10_O	18.71	87.15	35.33	27.92
	1-Cyclohexene-1-carboxaldehyde	C_10_H_16_O	22.91	21.59	9.51	18.51
	3-Hexenal	C_6_H_10_O	7.12	47.15	–	16.97
	Citral	C_10_H_16_O	26.59	58.42	64.34	56.96
	5-Ethylcyclopent-1-enecarboxaldehyde	C_8_H_12_O	16.00	42.81	12.36	5.01
	Benzaldehyde	C_7_H_6_O	19.52	84.37	58.97	56.48
	(E)-Hexadec-2-enal	C_16_H_30_O	26.96	–	19.19	–
	(E)-2-Octenal	C_8_H_14_O	16.24	–	102.83	55.21
	3-Furaldehyde	C_5_H_4_O_2_	17.75	–	72.84	44.06
	(E,E)-2,4-Nonadienal	C_9_H_14_O	24.17	19.27	32.37	20.40
	5-Methyl-2-furancarboxaldehyde	C_9_H_12_O	21.35	–	40.04	33.00
	(E)-2-Hexenal	C_6_H_10_O	9.35	–	1794.73	1673.09
	2-Hydroxy-benzaldehyde	C_9_H_10_O	20.48	–	5.49	–
	10-Undecenal	C_11_H_20_O	26.91	–	–	4.11
	(E)-2-Dodecenal	C_12_H_22_O	15.26	–	2.41	–
	(E)-Hexadec-2-enal	C_16_H_30_O	26.70	–	15.53	6.57
	(E)-2-Octenal	C_8_H_14_O	16.20	–	–	47.77
	4-Ethyl-benzaldehyde	C_9_H_10_O	25.48	–	–	2.92
	Hexanal	C_6_H_12_O	5.60	2.01	–	252.61
	(Z)-3,7-Dimethyl-2,6-octadienal	C_10_H_16_O	24.59	–	–	29.12
	2,4-Dimethyl-benzaldehyde	C_9_H_10_O	26.20	–	2.91	–
	5-Hydroxymethylfurfural	C_6_H_6_O_3_	36.49	–	479.25	249.20
Esters	5,6,7,7a-Tetrahydro-4,4,7a-trimethyl-2(4H)-benzofuranone	C_11_H_16_O_2_	43.08	3.27	3.09	–
	3,7-Dimethyl-1,6-octadien-3-ol formate	C_11_H_18_O_2_	7.52	1.63	–	–
	Formic acid, octyl ester	C_12_H_20_O_3_	21.01	12.39	9.23	–
	Salicylic acid ethyl ester	C_9_H_10_O_3_	28.83	17.54	16.49	–
	Methyl salicylate	C_8_H_8_O_3_	27.62	125.38	218.61	192.35
	(5Z)-6,10-Dimethyl-5,9-undecadiene-2-one	C_13_H_22_O	30.40	–	33.30	60.43
	1,5-Dimethyl-1-vinyl-4-hexenyl butyrate	C_14_H_24_O_2_	20.71	–	–	19.13
	Acetic acid, methyl ester	C_3_H_6_O_2_	13.30	–	–	6.47
	Terpinyl Acetate	C_12_H_20_O_2_	25.55	–	–	9.28
	Formic acid, cis-4-methylcyclohexyl ester	C_8_H_14_O_2_	15.45	44.58	–	–
Others	2,3-Dihydro-2-methyl-benzofuran	C_9_H_10_O	32.14	204.24	76.43	100.21
	2-Pentyl-furan	C_9_H_14_O	9.75	22.46	36.43	24.20
	2,3-Dihydro-benzofuran	C_8_H_8_O	40.12	6.75	–	6.51
	2-Ethyl-furan	C_6_H_8_O	15.56	–	7.21	–
	3-Furanmethanol	C_5_H_6_O_2_	24.57	–	–	27.34
	2,5-Furandicarboxaldehyde	C_6_H_4_O_3_	33.84	–	34.92	7.21
	Hexanoic acid	C_6_H_12_O_2_	30.21	–	20.81	29.23
	Acetic acid	C_2_H_4_O_2_	17.94	–	15.41	55.10
	2-Ethoxy-benzoic acid	C_8_H_14_O	28.43	–	3.11	–
	Formic acid	CH_2_O_2_	19.36	–	–	19.86
Total Content (µg kg^-1^)				3232.47	5489.75	4398.39

“-” indicates that the compounds were not detected.

**Figure 10:**
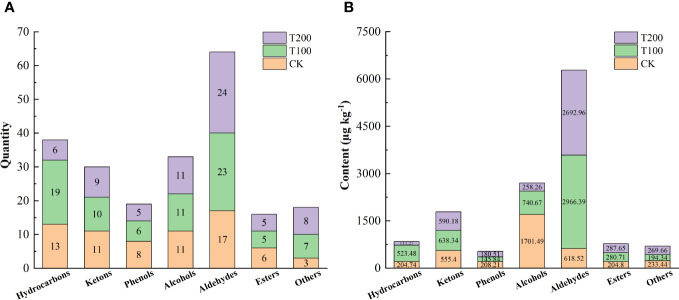
Effects of exogenous ALA on the quantity **(A)** and content **(B)** of volatile compounds in tomato fruit at maturity stage.

Different concentrations of ALA treatment could increase the number and content of various volatile substances to varying degrees (**Figure 10**). Among them, T100 treatment could significantly increase the number and content of hydrocarbons and aldehydes volatile substances compared with the control, and T200 significantly increased the number and content of aldehydes volatile substances. The characteristic volatile substances identified in tomato fruit in this study was presented in **Table 3**. A total of eight characteristic volatile substances were identified across the three treatments, namely 6-Methyl-5-hepten-2-one, 1-Penten-3-one, β-Ionone, 1-(2,6,6-Trimethyl-1,3-cyclohexadien-1-yl)-2-buten-1-one, 3-Hexenal, (E)-2-Hexenal, Hexanal and Methyl salicylate. According to the aroma types, it could be divided into irritating, floral, fruity and green aroma types. ALA treatments significantly increased the content of seven characteristic aroma substances except 3-Hexenal.

**Table 3:** Effects of exogenous ALA on characteristic volatile compounds content in tomato fruit at maturity stage.

Characteristic aroma components	Aroma type	Content (µg kg^-1^)		
		CK	T100	T200
6-Methyl-5-hepten-2-one	Fruity	121.53 ± 3.32 c	149.30 ± 0.82 a	188.57 ± 7.84 b
1-Penten-3-one	Pungent	34.84 ± 6.12 a	–	–
β-Ionone	Floral	45.64 ± 9.21 b	68.40 ± 4.23 a	48.56 ± 3.09 b
1-(2,6,6-Trimethyl-1,3-cyclohexadien-1-yl)-2-buten-1-one	Fruity	13.00 ± 2.85 b	12.85 ± 1.02 b	19.02 ± 0.53 a
3-Hexenal	Fruity	47.15 ± 2.23	–	16.97 ± 0.99
(E)-2-Hexenal	Fruity	–	1794.73 ± 53.77 a	1673.09 ± 54.13 b
Hexanal	Green	2.02 ± 0.03 b	–	252.61 ± 5.49 a
Methyl salicylate	Green	125.38 ± 2.25 c	218.61 ± 9.95 a	192.35 ± 1.17 b

"-” indicates that the compounds were not detected. Different lowercase letters indicate significant differences (P< 0.05).

## 4 Discussion

Soluble sugars, organic acids, and volatile components play a significant role in determining the flavor quality of fruits (Jin et al., 2023). Previous studies have shown that in peach fruit, the application of exogenous ALA can enhance the fruit’s soluble sugar content and sugar acid ratio (Ye et al., 2017). In our study, we found that the application of exogenous ALA significantly increased the content of sugar components and total soluble sugar in various tissues of tomato fruit, particularly in the flesh and septum tissues. Among the three tissues, glucose and fructose had the highest content, while sucrose had the lowest content, which aligns with the findings reported in the research on Tamarillo (Hu et al., 2023). Sucrose hydrolysis plays a crucial role in fruit development, involving various enzymes and genes. This process alters the composition of soluble sugars, consequently affecting the sweetness of fruits (Hou et al., 2020; Dou et al., 2022). Sucrose can be converted into fructose and UDP-glucose by SS, or fructose and glucose by AI and NI. SPS and SS can catalyze fructose and fructose-6-phosphatase (F-6-P) to synthesize sucrose, respectively (Hongxia et al., 2022; Varucha et al., 2022). Li et al. (Li et al., 2021) observed a positive correlation between the expression of *SS* and the sugar-acid ratio of tomato fruit. Up-regulation of *SS* expression contributed to the accumulation of glucose and fructose in the fruit. By silencing *INVINH1* (the inhibitor of the acid invertase gene Lin5), Jin et al. (Jin et al., 2009) found that tomato invertase activity could be increased, leading to an increase in fruit hexose content. Similarly, Wang et al. (Wang B, et al., 2021) obtained similar results by knocking out *INVINH1* in tomato, providing evidence that invertase is a key enzyme in sucrose metabolism. Zhang et al. (Zhang et al., 2022) discovered that overexpression of *SlSPS* significantly increased SPS activity, promoting sucrose synthesis and increasing sucrose content. Conversely, knockout of *SlSPS* showed the opposite results. In our study, we observed higher activities of AI and NI in different tissues of tomato fruits treated with exogenous ALA compared to the control group at both the mature green stage and breaker stage. Additionally, the ALA treatments significantly up-regulated the expression of *SlAI* and *SlNI*, enhanced the activity of SS-C in flesh, increased the expression level of *SlSS* in flesh, and decreased the activity of SPS in flesh. These changes resulted in a significant accumulation of fructose and glucose in the flesh. Previous studies have indicated that sucrose-accumulating cherry tomatoes exhibit higher levels of SS in the early stages of fruit development, suggesting that SS played a crucial role in the hydrolysis of sucrose during the early stages (Sun et al., 2022). In our study, we observed a decrease in the activity of SS-C in the fruit septum when treated with exogenous ALA during the breaker stage of tomato. Furthermore, the activity of SPS and the expression level of *SlSPS* decreased in both the flesh and septum, indicating a reduction in sucrose synthesis in these tissues. Interestingly, the sucrose content actually increased under ALA treatment during this stage, suggesting a potential link to sucrose transport (Ma et al., 2017; Durán-Soria et al., 2020). Moreover, we found that the activity of AI increased treated with exogenous ALA in the pulp, columella, and septum during the tomato maturity stage. It also up-regulated the expression of *SlAI*, leading to higher levels of glucose and fructose. Additionally, the activity of NI, SS-C, and SPS decreased, along with the expression of *SlNI*, *SlSS*, and *SlSPS*, resulting in a decrease in sucrose content. The decrease in NI and SS-C activity at this stage suggests that sucrose decomposition relied on AI. These findings align with previous research on cherry tomatoes, where exogenous auxin treatment inhibited ethylene production, SPS, and sucrose synthase (SUS) activity, down-regulated the expression of *SPS1*, *SPS4*, and *SUS3*, enhanced AI activity, decreased sucrose content, and increased fructose and glucose content (Tao et al., 2022).

Organic acids, as the primary carbon metabolism intermediates in plants, play a crucial role in influencing the taste and sensory quality of fruits (Liu R, et al., 2016). Previous studies have indicated variations in the content of organic acid components across different tissues of tomatoes. The chamber tissue predominantly contained a higher content of organic acid components compared to the flesh (Mounet et al., 2009). In our study, the accumulation of acids in flesh tissue was the least, except for quinic acid and malic acid, while it was the highest in columella tissue. Among the organic acids, citric acid was found to be the most abundant in tomato fruits, followed by malic acid, which was consistent with the results of previous transcriptome analysis of tomato fruits (Li et al., 2021). Additionally, our study showed that the application of exogenous ALA significantly reduced the contents of citric acid, quinic acid, shikimic acid, and oxalic acid in mature tomato fruits. This finding was consistent with the results reported by Wang et al. (Wang et al., 2022), suggesting that exogenous ALA spraying has a positive impact on the metabolism of organic acids in fruits. However, the accumulation and metabolism of organic acids in fruits involve a complex physiological and biochemical process. This process is regulated, synthesized, transported, and decomposed by various enzymes and genes (Quinet et al., 2019). Liao et al. (Liao et al., 2022) conducted a transcriptome analysis and discovered that PEPC, MDH, ME, CS, IDH, and ACO are involved in organic acid metabolism in litchi. The expression of *PEPC* and ACO aligned with the changed in citric acid content, while the expression of *MDH* correlates with the changes in malic acid content. In Arabidopsis thaliana, the highly expressed genes *PPC1* and *PPC2* were found to encode PEPC. When PPC1/PPC2 double mutants were created, a decrease in PEPC activity and a weakening of malic acid and citric acid synthesis were observed (Shi et al., 2015). Carine Guillet et al. (Guillet et al., 2002) specifically identified *PPC2* expression in tomato fruit. Increased PPC2 activity leads to an increase in PEPC activity as well as the content of malic acid and citric acid. Jia et al. (Jia et al., 2023) discovered that kiwifruit increased the activity of CS and MDH, up-regulated the expression of *AeCS2* and *AsMDH2*, and accumulated citric acid and malic acid. In our study, ALA treatments enhanced the activity of CS and PEPC, up-regulated *SlCS* and *SlPPC2* expression, reduced ACO activity, and up-regulated *SlACO* expression, thus promoting the accumulation of citric acid in different tissues of fruits at mature green stage. The activity of MDH was enhanced by ALA treatment, leading to an increase in the expression of *SlMDH1* and a significant rise in malic acid content in the flesh during at mature green stage. This finding was consistent with the study conducted by Yao et al. (Yao et al., 2011). The content of malic acid showed a positive correlation with MDH and *SlMDH* during the early developmental stage of tomato. During the breaker stage, due to the enhanced activity of ME, the expression level of *SlME1* increased, the malic acid was decarboxylated to pyruvate, causing a sharp decrease in the content of malic acid. In addition, PEPC activity and *SlPPC2* expression level increased under ALA treatment. This could be attributed to the conversion of PEP to oxaloacetate catalyzed by PEPC in the tricarboxylic acid cycle, which was also associated with the substrate of malate metabolism (Liu R, et al., 2016). In this study, ALA treatment increased the activity of PEPC and CS in various tissues. It also up-regulated the expression of *SlPPC2* and *SlCS*, while decreasing the activity of ACO and down-regulating the expression of *SlACO*. As a result, there was a decrease in the content of citric acid. The findings suggest that ALA may decrease the citric acid content in fruit by inhibiting ACO activity and reducing *SlACO* expression, thereby weakening the citric acid synthesis pathway. This was in line with the findings of Liu et al. (Liu et al., 2007). Additionally, it was possible that treating the fruit with exogenous ALA slowed down the TCA cycle during the maturity, leading to a lower citric acid content compared to the control group. Quinet et al. (Quinet et al., 2019) suggested that high levels of citric acid can inhibit ACO activity, thereby slowing down the TCA cycle. Other studies have indicated that ACO activity has minimal effect on citric acid content, with the main role being played by isocitrate dehydrogenase (IDH) (Tang et al., 2010). Whether this holds true in the current study requires further verification.

As one of the important sensory quality components of tomato fruit, aroma also affects consumers' choice of goods (Wu Q, et al., 2018). Volatile substances that could be classified into two states: free and bound in tomatoes. Free volatile substances could be detected by the human body, affecting the taste perception of acidity and sweetness (Goff and Klee, 2006). Bound volatile substances in tomatoes were present in the form of glycoside-bound compounds, such as trans-2-hexenal, α-ionone, guaiacol, and methyl salicylate, which were linked to pyranose through α-glycosidic bonds or bonded to diglycosides (Ortiz-Serrano and Gil, 2007). At present, there have been a lot of studies on the important volatile components and synthesis pathways in tomato fruits. It mainly originated from fatty acids, carotenoids, phenylalanine and branched-chain amino acids (Rodrigues Magalhães et al., 2023). In our study, a total of 114 volatile substances were detected, of which 69, 81 and 68 were detected in the control, T100 and T200, respectively. ALA treatments significantly increased the content of volatile substances in tomato fruit. There are 16 kinds of substances that had important contributions to the aroma in tomato fruits, which were cis-3-hexenal, hexanal, trans-2-hexenal, 3-methylbutanal, 2-methylbutanal, trans-2-heptenal, 2-phenylacetaldehyde, β-ionone, β-damascenone, 1-penten-3-one, 6-methyl-5-hepten-2-one, cis-3-hexenol, 2-phenylethanol, 3-methylbutanol, 2-isobutylthiazole, 1-nitro-2-ethylbenzene and methyl salicylate (Wang et al., 2015). In our study, we identified a total of eight characteristic volatile compounds: seven in the control group, five in the T100 group, and seven in the T200 group. Importantly, treatment with ALA significantly increased the concentration of these characteristic volatile compounds. The highest content was trans-2-hexenal, which was not detected in the control treatment. The highest content in the control was 2-cyclohexen-1-ol, and the content of cis-3-hexenal in the control treatment was higher than that in the T200 treatment. It was found that trans-2-hexenal was isomerized from cis-3-hexenal (Quinet et al., 2019). It has been reported that lipoxygenase C (TomloxC) and hydroperoxide lyase (HPL) catalyze linoleic acid or linolenic acid to form hexanal and cis-3-hexenal (Yilmaz et al., 2001). We considered that that ALA treatment increased the related enzymatic reaction in this process, increased the content of trans-2-hexenal and decreased the content of cis-3-hexenal. Chen et al. (Chen et al., 2022) found that (E) -2-hexenal isomerase (HI) could catalyze the conversion of (Z) -3-hexenal to (E) -2-hexenal. The content of 6-methyl-5-hepten-2-one, geranylacetone, β-ionone and β-damascenone, which are derived from carotenoids and give tomato fruit flavor and aromatic flavor, increased under ALA treatment, which may be ALA as a prerequisite for plant pigment synthesis. The accumulation of carotenoids in tomato fruit increases the content of aromatic substances based on it, which is consistent with the previous research results (Wang J, et al., 2021). Ortiz-Serrano et al. (Ortiz-Serrano and Gil, 2009) proposed that 6-methyl-5-hepten-2-one was a glycoside-bound volatile flavor substance, and its content increases with tomato ripening. Methyl salicylate and guaiacol were two types of substances that were catalyzed by phenylalanine through phenylalanine ammonia lyase (PAL) to form anti-cinnamic acid. It was produced by the action of salicylic acid methyl transferase 1 (SlSAMT1) and catechol-O-methyl transferase 1 (CTOMT1) encoded by *SlSAMT1* and *CTOMT1* genes respectively (Rambla et al., 2014; Hilton et al., 2021). Mageroy et al. (Mageroy et al., 2011) found that *CTOMT1* overexpression could increase the content of guaiacol. In this study, the content of methyl salicylate in the control fruit was higher than that in the ALA treatment, and the guaiacol with medicinal flavor was only detected in the control and T200 treatment. The content of guaiacol in the control fruit was 2.3 times that in the T200 treatment fruit.

**Figure 11:**
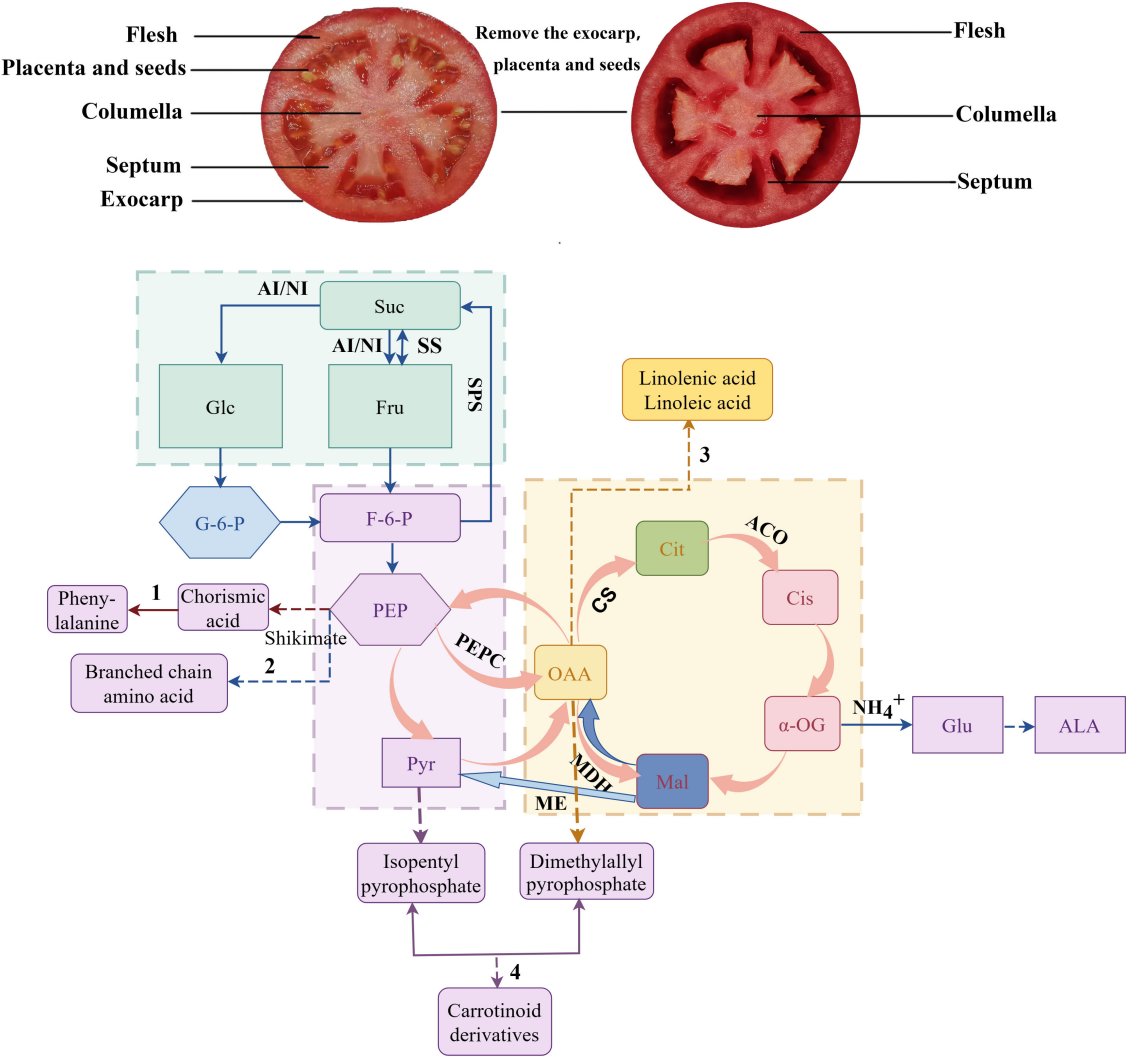
Correlation diagram of sugar acid metabolism and volatile substances in tomato fruit (by Figdraw). The straight line in the figure indicates that the downstream material is directly generated, and the dotted line indicates that the intermediate product is omitted. 1 is the phenylalanine synthesis pathway. 2 is the branched-chain amino acid synthesis pathway. 3 is the fatty acid synthesis pathway. 4 is the carotenoid derivatives synthesis pathway. Suc, sucrose; Glu, glucose; G-6-F, 6-phosphate-fructose; G-6-G, 6-phosphate-glucose; PEP, Phosphoenolpyruvate; Pyr, pyruvic acid; OAA, oxaloacetic acid; Mal, malic acid; Cit, citric acid; α-OG,α-ketoglutaric acid; Cis, isocitric acid; Glu, Glutamic acid; ALA, 5-aminolevulinic acid; SS, sucrose synthase (decomposition direction); SPS, sucrose phosphate synthase; AI, acid invertase; NI, neutral invertase; PEPC, phosphoenolpyruvate kinase; CS, citrate synthase; ACO, aconitase; ME, malic enzyme; MDH, malate dehydrogenase.

In summary, the application of 200 mg L^-1^ ALA solution at tomato fruit setting stage could significantly increase the content and type of aldehyde volatile substances in fruits. In addition, ALA treatments could significantly increase the accumulation of sugars in fruit flesh and septum tissues by regulating the activity of key enzymes and the expression of key genes in sugar and acid metabolism during fruit development, reduce the content of organic acids in various tissues, increase the ratio of sugar to acid in fruit, and improve the sugar and acid quality of tomato fruit (**Figure 11**).

## Data availability statement

The original contributions presented in the study are included in the article/supplementary material. Further inquiries can be directed to the corresponding authors.

## Author contributions

RL: Methodology, Visualization, Writing – original draft, Writing – review & editing. JW: Methodology, Visualization, Writing – review & editing. HY: Methodology, Visualization, Writing – review & editing. YN: Methodology, Visualization, Writing – review & editing. JS: Methodology, Writing – review & editing. QT: Methodology, Writing – review & editing. YW: Conceptualization, Funding acquisition, Resources, Supervision, Writing – review & editing. JY: Conceptualization, Funding acquisition, Resources, Writing – review & editing. ZT: Supervision, Visualization, Writing – review & editing. XX: Supervision, Visualization, Writing – review & editing. JX: Supervision, Writing – review & editing. LH: Supervision, Writing – review & editing. ZL: Supervision, Writing – review & editing. WL: Conceptualization, Funding acquisition, Resources, Supervision, Writing – review & editing.
